# An Interobserver Comparison of the Ultrasound Lexicon Classification of Thyroid Nodules: A Single-Center Prospective Validation Study

**DOI:** 10.3390/jcm14041222

**Published:** 2025-02-13

**Authors:** Ender Uysal, Burak Yangoz, Mustafa Sagan, Ismet Duman, Ahmet Sukru Alparslan

**Affiliations:** Department of Radiology, Health Sciences University Antalya Training and Research Hospital, 07100 Antalya, Turkey; enderun73@gmail.com (E.U.); saganmustafa@gmail.com (M.S.); behran27@gmail.com (I.D.); ahmetalprsln@gmail.com (A.S.A.)

**Keywords:** thyroid nodules, ultrasound imaging, risk stratification systems, interobserver agreement

## Abstract

**Background/Objectives**: Guidelines for the risk stratification of thyroid nodules are based on certain well-recognized sonographic features of nodules. However, significant variations in reported sensitivity and specificity values are observed due to the overlap of imaging characteristics between benign and malignant nodules. Additionally, differences in ultrasound (US) equipment and the varying experience levels of radiologists performing the imaging procedures contribute to these discrepancies. Inevitably, there are also interobserver differences. The aim of this study was to investigate interobserver agreement on these criteria using the international thyroid imaging reporting and data system (I-TIRADS) thyroid evaluation framework, independently assessed by three residents and one consultant. **Methods**: We included 393 patients who underwent ultrasound-guided fine needle aspiration biopsy (FNAB) within four months. In each case, longitudinal and transverse video images of the thyroid gland, neck chain, and biopsied nodules were recorded. The evaluations of the parameters defined in the I-TIRADS dictionary were then performed by a radiologist with 15 years of experience and radiology assistants with 3, 3, and 2 years of experience, respectively, blinded to the images, pathology data, and patient demographics. The parameters evaluated included composition, echogenicity, margin, direction of growth, calcification, extension beyond the thyroid, and lymph node. An interobserver comparison between the US lexicon classifications of thyroid nodules was then performed. **Results**: The results of our study showed that the highest level of consensus was observed in the ‘mixed predominantly cystic’ classification, indicating a solid consistency between the assessors (κ = 0.729). Conversely, the subcategories ‘Solid’, ‘Mixed Predominantly Solid’ and ‘Spongiform’ showed moderate agreement, while the “Pure Cyst” subcategory exhibited the lowest level of agreement among the assessors (κ = 0.292). Agreement among the three radiology assistants was strong concerning the evaluation of nodule composition, growth direction, and lymph node assessment. In contrast, a moderate level of consensus was noted regarding the assessment of extrathyroidal extension, margins, and echogenicity. Notably, the parameter exhibiting moderate agreement across all readers was the presence of echogenic foci or calcifications. **Conclusions**: the reproducibility observed in the parameters defined within the lexicon supports its potential to enhance consistency and interobserver agreement in thyroid nodule assessment.

## 1. Introduction

In the analysis of the GLOBOCAN 2020 data, the rate per 100,000 population was 10.1 for women and 3.1 for men. This indicates that thyroid cancer is significantly more common in women [1]. However, the mortality rate was found to be relatively low, with female patients demonstrating a rate of 0.5 per 100,000 and male patients exhibiting a rate of 0.3 per 100,000. Thyroid ultrasound is a widely used diagnostic tool for the discrimination of benign nodules from malignant nodules, in addition to its role in guiding fine needle aspiration cytology in cases of suspicious nodules [2,3]. The utilization of fine-needle aspiration biopsy (FNAB) has been demonstrated to yield a diagnostic yield of 9.2–14.8% for malignancies identified via clinical examination [4,5,6]. The ultrasonography (US) features that have been shown to be suggestive of malignancy include the presence of microcalcifications, hypoechogenicity, irregular margins, the absence of halos, a predominantly solid composition, and intranodular vascularity [4,5,6,7].

However, sensitivity and specificity values vary in the literature due to the overlap between benign and malignant nodules, the use of different US equipment, the difference in experience between the radiologists performing the imaging, and inevitably interobserver differences [4,6,8,9]. Furthermore, the increase in incidental thyroid nodules detected using imaging modalities such as US, computed tomography (CT), magnetic resonance imaging (MRI) scans, and 18F-fluorodeoxyglucose (FDG)-positron emission tomography (PET)/CT scans highlights the importance of appropriate clinical management of these patients [10,11,12,13,14,15,16,17]. In clinical management, it is crucial to recognize the different characteristics of thyroid nodules and patient-specific risk profiles. Therefore, over the last two decades, many organizations have developed ultrasound-based risk stratification systems (RSSs) and management guidelines for thyroid nodules [2,18,19,20,21,22,23]. These classification systems aim to identify the risk of malignancy and guide management while establishing a standardized language for communication and reporting. However, estimated cancer risk and management recommendations for a nodule may vary depending on the system used, which can lead to confusion among clinicians and patients [24].

In light of the aforementioned information, Durante et al. tried to develop a lexicon and atlas of US descriptors for thyroid nodules using I-TIRADS [25]. In the present study, observers were tasked with the evaluation of descriptors within seven distinct categories, namely (1) composition, (2) echogenicity, (3) margin, (4) shape, (5) echogenic foci, (6) extrathyroidal extension, and (7) lymph nodes. The objective of this study was to investigate the interobserver agreement of these criteria using the I-TIRADS thyroid evaluation criteria, evaluated separately by three residents and one consultant.

## 2. Materials and Methods

This study was conducted in accordance with the Declaration of Helsinki and approved by the Ethics Committee of Antalya Training and Research Hospital (approval no: No: 2448/dated 25 June 2019). In this prospective study, patients were prospectively followed up from September 2023 to January 2024, and informed consent was obtained. US-guided FNABs were performed in 441 thyroid nodules in 441 patients. The risk stratification system (RSS) for the I-TIRADS lexicon has not yet been officially defined. However, several parameters outlined in the lexicon, such as echogenicity, calcifications, and margin characteristics, have been widely discussed in the literature regarding their sensitivity and specificity in distinguishing benign from malignant thyroid nodules [3,4,6,8]. Some of these features are also utilized in existing RSS classifications [18,19,20]. In our study, biopsy decisions were based on the parameters explicitly defined in the I-TIRADS lexicon [25]. Each nodule was classified according to the I-TIRADS criteria described by Durante et al., with particular attention to lexicon-defined ultrasound characteristics, and no additional sources were considered [25]. All FNABs were performed under ultrasound guidance using an 18G fine needle, ensuring appropriate sterilization protocols, and conducted by experienced interventional radiologists. The demographic data of all patients were obtained from the automation system of our hospital. Image quality was assessed by the expert radiologist of this study before the grading process began. Patients included in this study met the following criteria: they were over 18 years of age, had ultrasound images of sufficient quality for diagnostic evaluation, and had no history of prior thyroid surgery, ablation, or embolization therapy. Patients with inadequate imaging quality or a history of interventional thyroid procedures were excluded to ensure consistency and reliability in the assessment. Finally, 393 patients (86 men and 307 women; mean age, 53 years; age range, 16–80 years) with 393 nodules were enrolled in this study.

**Figure 1:**
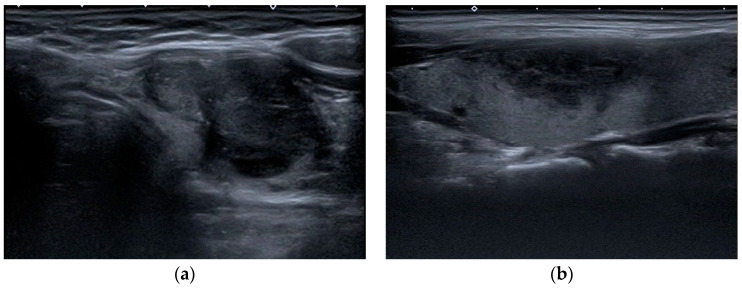
On the left side of the image (**a**), a markedly hypoechoic solid nodule is localized in the left lobe of the thyroid gland, categorized as solid and hypoechoic compared to the surrounding scalene muscles. Postoperative pathology confirmed the diagnosis of papillary carcinoma. On the right side of the image (**b**), a sagittal section of an irregularly bordered nodule with malignant features localized in the left lobe of the thyroid gland is shown. The FNAB result classified the nodule as Bethesda Category V.

**Figure 2:**
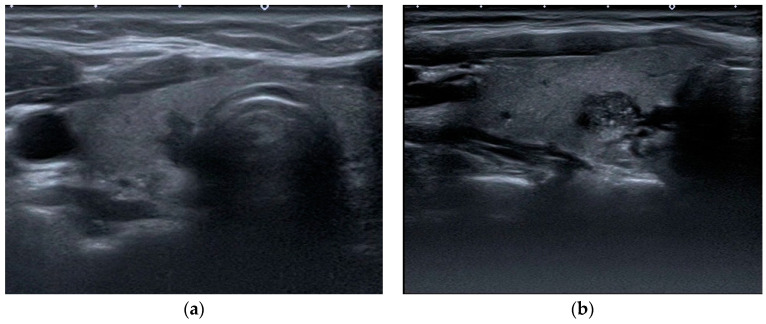
A subcentimetric nodule localized in the right lobe of the thyroid gland is depicted on the left side (**a**), demonstrating a taller-than-wide appearance, with postoperative pathology confirming the diagnosis as papillary carcinoma. On the right side (**b**), the image presents a sagittal section of a nodule localized in the right lobe of the thyroid gland, showing microcalcifications on gray-scale ultrasound imaging. FNAB result classified this nodule as Bethesda Category IV.

**Figure 3:**
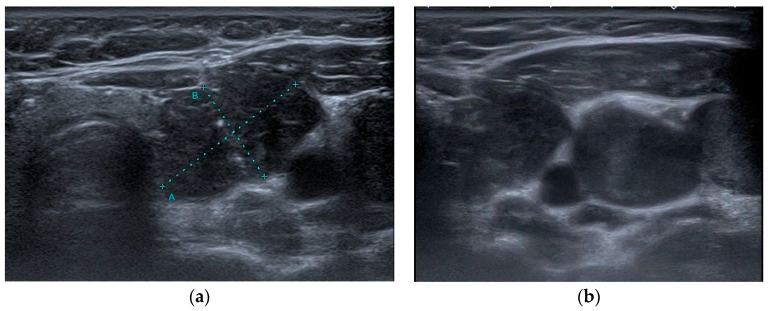
On the left (**a**), a gray-scale transverse ultrasonographic image shows an irregularly contoured lesion in the left thyroid lobe, measuring 26 × 17 mm in its largest dimension (A: length, B: width), extending beyond the thyroid capsule into the left anterolateral neck, obliterating the lateral carotid space and anterior scalene muscle planes. FNAB classified the lesion as Bethesda Category VI, with postoperative pathology confirming papillary thyroid carcinoma. On the right (**b**), a markedly hypoechoic nodule with irregular margins is observed in the left thyroid lobe, accompanied by two adjacent nodular metastatic lymph nodes in the left cervical chain with obliterated fatty hilum, classified as suspicious. Postoperative pathology confirmed papillary carcinoma invasion in both lymph nodes.

US examinations were performed on a Toshiba Aplio 500 (Canon Medical Systems, Tokyo, Japan) equipped with a high-frequency 7.5 MHz linear array transducer. In each case, longitudinal and transverse video images of the whole thyroid gland, the neck chain, and biopsied nodules were recorded. After recording, patient data were removed from videos and images. A radiologist with 15 years of experience and radiology residents with 3, 3, and 2 years of experience, respectively, who were blinded to the pathology data and patient demographics, participated in this study with the following objectives. Prior to the assessment, all assessors were provided with the relevant I-TIRADS lexicon article prepared by Durante et al. and allocated sufficient time for familiarization and review [25]. The video images of the entire thyroid parenchyma, neck chain, and biopsied nodule were sent to each evaluator via cloud storage. The evaluations of the parameters defined in the I-TIRADS lexicon (composition, echogenicity, margin, direction of growth, calcification, extension beyond the thyroid, and lymph node) were recorded independently from each other. All assessments were requested from the observers to be completed within 4 months. The retrospective assessment of the ultrasonographic features of the nodules was followed by their classification according to the I-TIRADS Lexicon. The internal content of a nodule was categorized as solid, mixed predominantly solid, mixed predominantly cystic, spongiform, or pure cystic (Figure 1). The echogenicity of the nodule was categorized as hypoechogenic (marked or mild), isoechogenic, hyperechogenic, and anechoic, with the predominant echogenicity being referenced against that of the normal thyroid gland and anterior neck muscle (Figure 1). The margin of the nodule was categorized as smooth, irregular, or ill defined (Figure 1). The direction of growth was classified as wider-than-tall or taller-than-wide (Figure 2). Calcification was classified as microcalcification, macrocalcification, rim calcification, or echogenic foci with comet-tail artifacts (Figure 2). Extrathyroidal extension was evaluated as gross extrathyroidal extension, suspicious minor extrathyroidal extension, or capsule contact (Figure 3). Lymph nodes were categorized as suspicious, indeterminate, or nonsuspicious lymph nodes (Figure 3).

Statistical analyzes in this study were performed using the SPSS 27.0 (IBM Inc., Chicago, IL, USA) program. The Kolmogrov-Smirnov test, histogram analysis, skewness/kurtosis data, and Q-Q plots were used to evaluate the assumptions of a normal distribution. Qualitative parameters are expressed as frequency and percentage (%). The descriptive statistics of the numerical and categorical American Thyroid Association (ATA) obtained in this study were analyzed and expressed as IQR (median [minimum–maximum]) since the quantitative parameters did not exhibit a normal distribution pattern. Relationships between the two groups were examined with the Mann–Whitney U test. Relationships between nominal parameters were evaluated with either chi-square analysis or Fisher’s exact tests. The agreement between the interobservers and the reliability of decisions was determined using Fleiss kappa analysis. In the entire study, the type-I error rate was taken as 5% (α = 0.05), and *p* < 0.05 was accepted as the significant limit.

## 3. Results

### 3.1. Study Population

The mean patient age was 53 (range, 16–80 years). The mean nodule size was 18 *mm* (mm) (range, 5–70 mm) (Table 1).

A statistical analysis revealed that female patients exhibited greater age values (*p* < 0.001), while male patients exhibited higher nodule sizes (*p* = 0.003), (Table 2). The number of multiple nodules was 79 (91.9%) in male patients and 273 (89.9%) in female patients, while the number of patients with a single nodule was seven (8.1%) and thirty-four (11.1%), respectively (*p* = 0.431). The number of patients with nodules larger than one centimeter (cm) was 81 (94.2%) in males and 276 (89.9%) in females, while the number of patients with nodules smaller than one cm was five (5.8%) and thirty-one (10.1%), respectively (*p* = 0.224), (Table 2).

### 3.2. Agreement for US Characteristic According to the I-TIRADS Lexicon

The US features were evaluated by assessing the interobserver agreements between all four observers, consisting of one radiology expert and three radiology residents. The interpretation and classification of Kappa values were carried out in accordance with Cohen’s (1961) recommendations, following objective and generally accepted literature suggestions for interpretation [26]. The results revealed a statistically substantial consensus across all evaluations. Notably, there was significant agreement on echogenicity, echogenic foci/calcifications, and extrathyroidal extension, with agreement levels at acceptable (fair) values (κ = 0.343, κ = 0.389, and κ = 0.333, respectively). When the evaluations of composition, margin, direction of growth, and lymph nodes were examined, moderate agreement was observed among the observers (κ = 0.538, κ = 0.431, κ = 0.481, and κ = 0.452, respectively). A detailed summary of interobserver agreement percentages and kappa values is provided in Table 3.

Table 4 presents the interobserver agreement among the three readers (residents) for the ultrasound features and the I-TIRADS levels. The agreement among the three radiology residents was good for nodule composition, direction of growth, and lymph node evaluation (κ = 0.728, κ = 0.657, and κ = 0.625, respectively). Conversely, a moderate degree of consensus was observed regarding the evaluation of extrathyroidal extension, margin, and echogenicity (κ = 0.571, κ = 0.585, and κ = 0.565, respectively). The only parameter with fair agreement was the echogenic foci/calcifications among all readers (κ = 0.391).

We conducted a detailed analysis of the decisions made by four observers and evaluated their interobserver agreement across various subgroups of sonographic measurements, as summarized in Table 5. The highest level of consensus was observed in the “mixed predominantly cystic” decision, indicating strong consistency (κ = 0.729). This elevated concordance may be attributed to being well defined in the lexicon, with clear and distinct imaging characteristics, reducing ambiguity during classification. Moderate agreement was found in the “Solid” and “Mixed Predominantly Solid” subgroups (κ = 0.552, κ = 0.498, respectively), while “Spongiform” demonstrated fair agreement (κ = 0.497). The “Pure Cyst” subgroup showed the lowest agreement (κ = 0.292).

For echogenicity, moderate agreement was achieved in “Markedly Hypoechoic” (κ = 0.514), whereas other subgroups, including “Mildly Hypoechoic”, “Isoechoic”, and “Anechoic”, exhibited fair agreement (κ = 0.352, κ = 0.349, and κ = 0.341, respectively). The lowest agreement was noted in the “Hyperechoic” subgroup (κ = 0.151).Regarding margins, the “Smooth” subgroup showed moderate agreement (κ = 0.511), while the “Irregular” and “Defined” subgroups had fair agreement (κ = 0.352, κ = 0.361, respectively). For direction of growth, both the “Taller-than-Wide” and “Wider-than-Tall” subgroups demonstrated moderate agreement (κ = 0.481).

In the evaluation of echogenic foci/calcifications, moderate agreement was observed in “Macrocalcifications” (κ = 0.532), while “Punctate Echogenic Foci” showed fair agreement (κ = 0.448). “Echogenic Foci with Comet-Tail Artifact”, “Peripheral (Rim) Calcifications”, and “Without Foci” had the lowest agreement levels (κ = 0.394, κ = 0.321, and κ = 0.275, respectively). For extrathyroidal extension, all subgroups demonstrated fair agreement. “Capsula Contact” showed the highest consistency (κ = 0.378), followed by “Gross Extrathyroidal Extension” and “Suspicious Minor Extrathyroidal Extension” (κ = 0.298, κ = 0.288, respectively).

Finally, in the evaluation of lymph nodes, moderate agreement was observed in “Nonsuspicious Lymph Nodes” (κ = 0.504) and “Suspicious Lymph Nodes” (κ = 0.483), while “Indeterminate Lymph Nodes” exhibited fair agreement (κ = 0.364).

These findings highlight variability in interobserver reliability across different subgroups, with κ values indicating agreement levels ranging from fair to strong. All results were statistically significant (*p* < 0.001).

## 4. Discussion

The results of our study showed that the highest level of consensus was observed in the ‘mixed predominantly cystic’ classification, indicating a solid consistency between the assessors. However, the subcategories ‘Solid’ and ‘Mixed Predominantly Solid’ showed moderate agreement, while the category ‘Spongiform’ also reflected moderate agreement. Higher agreement in these categories might be achieved if the boundaries between them were more clearly defined. On the other hand, the ‘Pure Cyst’ subcategory exhibited the lowest degree of agreement between the assessors. The low interobserver agreement for the “Pure Cyst” category (κ = 0.292) is likely due to the absence of a standardized definition in TIRADS lexicons, which leads to inconsistencies among observers [25]. Additionally, ultrasound artifacts and image quality issues, such as posterior acoustic enhancement and comet-tail artifacts, further complicate accurate classification [27]. Furthermore, some cystic nodules contain small solid components, leading to misclassification and overlap with other subcategories, highlighting the need for improved lexicon standardization [28]. Agreement among the three radiology assistants was strong concerning the evaluation of nodule composition, growth direction, and lymph node assessment. In contrast, a moderate level of consensus was noted regarding the assessment of extrathyroidal extension, margins, and echogenicity. This moderate interobserver agreement likely stems from the inherent subjectivity in interpreting these ultrasonographic features. For extrathyroidal extension, moderate agreement may result from difficulties in distinguishing true capsular invasion from perithyroidal tissue contact or compression artifacts. The absence of precise criteria for minor versus gross extrathyroidal extension in the I-TIRADS lexicon may also contribute to discrepancies. With regard to margins, the challenge lies in differentiating between smooth, irregular, and ill-defined borders, especially in cases with overlapping characteristics or suboptimal image quality. Echogenicity assessments are similarly subjective and are influenced by technical factors such as gain settings and operator experience. Moreover, the parameter exhibiting moderate agreement across all readers was the presence of echogenic foci or calcifications. This variability may be attributed to the small size and subtle appearance of these features, which can be easily overlooked or misclassified, especially in nodules with heterogeneous internal structures.

The I-TIRADS lexicon is emerging as a global consensus ultrasound dictionary that aims to provide consistent, sustainable, and objective communication between radiologists and clinicians [25]. An effective lexicon strives for high diagnostic accuracy and strong interobserver consistency, regardless of experience. Ultrasound is inherently subjective and user-dependent; however, guidelines developed through studies have enabled more objective evaluations and higher diagnostic accuracy for distinguishing benign from malignant thyroid lesions, as is the case for other organs and systems [22,25,29,30].

Numerous studies evaluating the diagnostic performance and interobserver consistency of the European TIRADS (EU-TIRADS), American College of Radiology TIRADS (ACR-TIRADS), Korean TIRADS (K-TIRADS), and Chinese TIRADS guidelines for thyroid ultrasound are available in the literature [18,27,29]. In our study, we aimed to investigate the differences in the evaluation of the I-TIRADS lexicon between observers [22,23,24,25,29,30].

Previous interobserver reliability studies have reported variable levels of agreement for ultrasonographic features defined in existing TIRADS classifications, such as composition, shape, calcifications, echogenicity, and margins [27,30,31,32]. It is known that interobserver agreement also varied greatly depending on the observers’ experience [27]. Moreover, well-defined ultrasonographic features in lexicons have been shown to improve interobserver agreement regardless of experience. On the other hand, insufficient definitions and the use of subjective criteria increase the rate of unnecessary invasive procedures and impose additional costs, thereby increasing the burden on the healthcare system [33,34,35].

A recent multicenter study reported insufficient interobserver agreement in the evaluation of nodule margins, whereas the agreement for microcalcifications was moderate. These findings contrast with earlier studies that reported higher levels of agreement. Additionally, the study revealed that smaller nodules showed better agreement in echogenicity assessments, while larger nodules exhibited greater agreement in identifying the presence of microcalcifications [32].

In our study, high interobserver consistency was observed across all parameters in the evaluation of thyroid nodules. Among the four evaluators, including a radiology expert with over 20 years of experience in thyroid assessments, moderate agreement was achieved for the parameters of composition, margin, direction of growth, extrathyroidal extension, and lymph node assessment. There was fair agreement in the evaluation of extrathyroidal extension, echogenicity, and calcification. Significant agreement was observed in all parameters, with the direction of growth being the most significant. These findings indicate that the I-TIRADS lexicon adequately ensures interobserver agreement, independent of experience. However, the results of our study do not surpass those achieved with previous TIRADS classifications. This raises questions about the I-TIRADS lexicon’s contribution to enhancing objectivity compared to the existing RSSs [27,30,36]. Although our study is single-centered and limited by a relatively small sample size, drawing definitive conclusions at this stage is premature. Larger, multicenter studies are necessary to clarify the contribution of the I-TIRADS lexicon to objective evaluation and interobserver agreement.

The comparison of the three radiology residents showed nominally stronger agreement than the analysis with the expert. There was good agreement on composition, growth direction, and lymph node parameters; moderate agreement on extrathyroidal extension, echogenicity, and margin parameters; and fair agreement on the calcification parameter. The higher agreement among the residents may stem from their shared education and training within the same institution. Additionally, all participating residents had completed their formal ultrasound training and were instructed to study the I-TIRADS lexicon before being included in this study. This preparatory approach may have contributed to the higher consistency observed among the residents. In contrast, some studies in the literature have reported reduced agreement levels between junior and senior observers due to variations in experience [37]. However, in our study, the groups including and excluding the expert yielded similar results, with the junior-only group demonstrating higher agreement across all parameters. This finding highlights the potential impact of standardized training and familiarity with specific lexicons in achieving consistent evaluations [38].

Regarding the subgroups, it was observed that the low level of agreement in hyperechoic parameter decreased the overall level of agreement in echogenicity evaluations. The low agreement in hyperechoic nodules suggests a need for more objective descriptors within the lexicon, as hyperechogenicity is highly dependent on machine settings and operator technique, leading to greater variability among observers [3,20,22]. This emphasizes the necessity for more precise and objective definitions within the I-TIRADS lexicon. In the lexicon, thyroid nodules without echogenic foci or calcifications are excluded from classification under the echogenic focus/calcification parameter [25]. However, it is well documented that nodules lacking echogenic foci or calcifications do exist, raising concerns about the comprehensiveness of this categorization [22,38,39]. The current version of I-TIRADS would benefit from the inclusion of additional subcategories such as “without foci” under the echogenic focus/calcification category. Improvements in categorization criteria may enhance the clarity and objectivity of the lexicon, ultimately aiding in better clinical decision-making.

In our analysis of subgroups, the parameters demonstrating the highest interobserver agreement included mixed predominantly cystic, solid composition, markedly hypoechoic echogenicity, smooth margins, macrocalcifications, and nonsuspicious lymph nodes. Conversely, parameters with the lowest levels of agreement were pure cyst, hyperechoic echogenicity, absence of foci, gross extrathyroidal extension, and suspicious minor extrathyroidal extension. These findings suggest that refining and providing more objective criteria for these parameters could enhance interobserver agreement. To validate the findings of our study, multicenter research with a larger and more diverse group of evaluators is required. Increasing both the number and the variability in the evaluators’ experience would generate higher-quality data, thereby improving the reliability of the lexicon and its application in clinical practice.

As highlighted in a 2024 meta-analysis, ACR-TIRADS, EU-TIRADS, and K-TIRADS categorize thyroid nodules quite differently. K-TIRADS tends to classify nodules as high risk, ACR-TIRADS as intermediate risk, and EU-TIRADS as low risk. This variation in categorization supports the observation that these RSSs exhibit differing behaviors when assessing thyroid nodules, particularly in malignancy risk estimation. The findings are critical for developing a unified international system like I-TIRADS to enhance consistency across clinical practices [40].

Moreover, advancements in artificial intelligence strategies are also being utilized in the evaluation of thyroid nodules. In a study conducted by Tong et al., it was suggested that an optimized artificial intelligence strategy for thyroid nodule management could reduce diagnosis time-related costs without compromising diagnostic accuracy for senior radiologists. However, it is noted that a traditional, fully artificial intelligence strategy may still be more beneficial for less experienced radiologists [41].

Our study has several limitations that need to be addressed. First, the relatively small number of nodules evaluated and the low rate of malignancy among nodules undergoing FNAB may have affected the evaluations. Second, the fact that all observers were from the same institution may have impacted the consistency of the assessments. To overcome these limitations, future studies should aim to adopt a multicenter approach. Increasing the number and diversity of cases can help to improve the effectiveness of the evaluations. Furthermore, the inclusion of additional case series from different centers, with varying levels of observer experience and ultrasound devices, may provide more comprehensive and reliable information.

## 5. Conclusions

Although different ultrasound parameters were evaluated, our interobserver agreement study yielded results consistent with those of studies on existing RSS classifications. It is noteworthy that various RSSs, such as EU-TIRADS, K-TIRADS, and ACR-TIRADS, assess thyroid nodules, particularly low-risk nodules, in differing ways. However, I-TIRADS offers a universal lexicon. The I-TIRADS lexicon aims to establish a globally standardized RSS for thyroid nodule evaluation, providing a unified framework for classification. The reproducibility observed in the parameters defined within the lexicon supports its potential to enhance consistency and interobserver agreement in thyroid nodule assessment.

## Figures and Tables

**Table 1:** Distribution characteristics of quantitative parameters in the general sample.

Parameter	Minimum	Maximum	Distribution ^†^
Age (years)	16	80	53 (16–80)
Nodule size (mm)	5	70	18 (5–70)

^†^ Parameters are expressed as IQR (interquartile range) [median, min, and max].

**Table 2:** Comparison of quantitative parameters by gender.

	Gender		*p*
	Male (n = 86, 21.9%)	Female (n = 307, 78.1%)	
	Distribution *		
Age (years)	59.5 (27–80)	83 (16–79)	<0.001 ^a^
Nodule size (mm)	23 (7–70)	17 (5–80)	0.003 ^a^
Number of multiple nodules	79(91.9%)	273 (89.9%)	
Number of solitary nodules	7(8.1%)	34(11.1%)	
Nodule size (categorized)			
<1 cm	5 (5.8%)	31 (10.1%)	0.224 ^b^
≥1 cm	81 (94.2%)	276 (89.9%)	

* Parameters are expressed as IQR (interquartile range) [median, min, and max]. ^a^ Mann–Whitney U test, ^b^ Pearson’s chi-squared test.

**Table 3:** Interobserver agreements among one radiology expert and three residents.

US Feature	Interobserver Agreement (%)	Κ	%95 CI	*p* Value
Composition	69.80%	0.538	0.511–0.593	<0.001
Echogenicity	55.26%	0.343	0.318–0.367	<0.001
Margin	77.78%	0.431	0.398–0.464	<0.001
Direction of growth	90.88%	0.481	0.441–0.521	<0.001
Echogenic foci/calcifications	58.78%	0.389	0.364–0.413	<0.001
Extrathyroidal extension	87.32%	0.333	0.298–0.368	<0.001
Lymph nodes	88.25%	0.452	0.419–0.485	<0.001

**Table 4:** Interobserver agreements among three radiology residents.

US Feature	Interobserver Agreement (%)	Κ	%95 CI	*p* Value
Composition	81.59%	0.728	0.691–0.765	<0.001
Echogenicity	73.54%	0.565	0.527–0.603	<0.001
Margin	83.12%	0.585	0.539–0.632	<0.001
Direction of growth	92.88%	0.657	0.600–0.715	<0.001
Echogenic foci/calcifications Foci/calcifications	77.44%	0.391	0.367–0.415	<0.001
Extrathyroidal extension	91.09%	0.571	0.521–0.622	<0.001
Lymph nodes	90.75%	0.625	0.578–0.671	<0.001

**Table 5:** Detailed analysis of the decisions of 4 radiologists (1 radiologist and 3 resident doctors) and their interobserver agreements according to subgroups among sonographic measurements.

US Feature	Subgroup	Κ	%95 CI	*p* Value
Composition	Solid	0.552	0.512–0.593	<0.001
	Mixed predominantly solid	0.498	0.457–0.538	<0.001
	Mixed predominantly cystic	0.729	0.689–0.77	<0.001
	Spongiform	0.497	0.456–0.537	<0.001
	Pure cyst	0.292	0.252–0.333	<0.001
Echogenicity	Markedly hypoechoic	0.514	0.474–0.554	<0.001
	Mildly hypoechoic	0.352	0.311–0.392	<0.001
	Isoechoic	0.349	0.309–0.389	<0.001
	Hyperechoic	0.151	0.11–0.191	<0.001
	Anechoic	0.341	0.3–0.381	<0.001
Margin	Irregular	0.352	0.311–0.392	<0.001
	defined	0.361	0.321–0.402	<0.001
	Smooth	0.511	0.471–0.552	<0.001
Direction of Growth	Taller-than-wide	0.481	0.441–0.521	<0.001
	Wider-than-tall	0.481	0.441–0.521	<0.001
Echogenic Foci/Calcifications	Punctate echogenic foci/microcalcifications	0.448	0.407–0.488	<0.001
	Macrocalcifications	0.532	0.491–0.572	<0.001
	Peripheral (rim) calcifications	0.321	0.281–0.362	<0.001
	Echogenic foci with comet-tail artifacts	0.394	0.354–0.434	<0.001
	Without foci	0.275	0.235–0.315	<0.001
Extrathyroidal extension	Gross extrathyroidal extension	0.298	0.257–0.338	<0.001
	Suspicious minor extrathyroidal extension	0.288	0.247–0.328	<0.001
	Capsula contact	0.378	0.338–0.419	<0.001
Lymph Nodes	Suspicious lymph node	0.483	0.442–0.523	<0.001
	Indeterminate lymph node	0.364	0.323–0.404	<0.001
	Nonsuspicious lymph node	0.504	0.464–0.545	<0.001

## Data Availability

The data supporting the findings of this study are not publicly available due to privacy and ethical restrictions. However, data may be available from the corresponding author upon reasonable request and subject to institutional approval.

## Abbreviations

The following abbreviations are used in this manuscript:

ATA	American Thyroid Association
ACR-TIRADS	American College of Radiology Thyroid Imaging Reporting and Data System
CI	Confidence Interval
CT	Computed Tomography
EU-TIRADS	European Thyroid Imaging Reporting and Data System
FDG-PET/CT	18F-Fluorodeoxyglucose Positron Emission Tomography/Computed Tomography
FNAB	Fine Needle Aspiration Biopsy
IQR	Interquartile Range
I-TIRADS	International Thyroid Imaging Reporting and Data System
K-TIRADS	Korean Thyroid Imaging Reporting and Data System
MRI	Magnetic Resonance Imaging
RSSs	Risk Stratification Systems
SPSS	Statistical Package for the Social Sciences
US	Ultrasonography

## References

[1] Pizzato M., Li M., Vignat J., Laversanne M., Singh D., La Vecchia C., Vaccarella S. (2022). The epidemiological landscape of thyroid cancer worldwide: GLOBOCAN estimates for incidence and mortality rates in 2020. Lancet Diabetes Endocrinol..

[2] Frates M.C., Benson C.B., Charboneau J.W., Cibas E.S., Clark O.H., Coleman B.G., Cronan J.J., Doubilet P.M., Evans D.B., Goellner J.R. (2006). Management of thyroid nodules detected at US: Society of Radiologists in Ultrasound consensus conference statement. Ultrasound Q..

[3] Marqusee E., Benson C.B., Frates M.C., Doubilet P.M., Larsen P.R., Cibas E.S., Mandel S.J. (2000). Usefulness of Ultrasonography in the Management of Nodular Thyroid Disease. Ann. Intern. Med..

[4] Papini E., Guglielmi R., Bianchini A., Crescenzi A., Taccogna S., Nardi F., Panunzi C., Rinaldi R., Toscano V., Pacella C.M. (2002). Risk of Malignancy in Nonpalpable Thyroid Nodules: Predictive Value of Ultrasound and Color-Doppler Features. J. Clin. Endocrinol. Metab..

[5] Nam-Goong I.S., Kim H.Y., Gong G., Lee H.K., Hong S.J., Kim W.B., Shong Y.K. (2003). Ultrasonography-guided fine-needle aspiration of thyroid incidentaloma: Correlation with pathological findings. Clin. Endocrinol..

[6] Khoo M.L.C., Asa S.L., Witterick I.J., Freeman J.L. (2002). Thyroid calcification and its association with thyroid carcinoma. Head Neck.

[7] Kim S.J., Kim E.K., Park C.S., Chung W.Y., Oh K.K., Yoo H.S. (2003). Ultrasound-Guided Fine-Needle Aspiration Biopsy in Nonpalpable Thyroid Nodules: Is It Useful in Infracentimetric Nodules?. Yonsei Med. J..

[8] Kim E.-K., Park C.S., Chung W.Y., Oh K.K., Kim D.I., Lee J.T., Yoo H.S. (2002). New Sonographic Criteria for Recommending Fine-Needle Aspiration Biopsy of Nonpalpable Solid Nodules of the Thyroid. Am. J. Roentgenol..

[9] Frates M.C., Benson C.B., Doubilet P.M., Cibas E.S., Marqusee E. (2003). Can Color Doppler Sonography Aid in the Prediction of Malignancy of Thyroid Nodules?. J. Ultrasound Med..

[10] Brander A., Viikinkoski P., Nickels J., Kivisaari L. (1991). Thyroid gland: US screening in a random adult population. Radiology.

[11] Ezzat S. (1994). Thyroid incidentalomas. Prevalence by palpation and ultrasonography. Arch. Intern. Med..

[12] Tomimori E., Pedrinola F., Cavaliere H., Knobel M., Medeiros-Neto G. (1995). Prevalence of Incidental Thyroid Disease in a Relatively Low Iodine Intake Area. Thyroid.

[13] Gnarini V.L., Brigante G., Della Valle E., Diazzi C., Madeo B., Carani C., Rochira V., Simoni M. (2013). Very high prevalence of ultrasound thyroid scan abnormalities in healthy volunteers inModena, Italy. J. Endocrinol. Investig.

[14] Youserm D.M., Huang T., A Loevner L., Langlotz C.P. (1997). Clinical and economic impact of incidental thyroid lesions found with CT and MR. Am. J. Neuroradiol..

[15] Yoon D.Y., Chang S.K., Choi C.S., Yun E.J., Seo Y.L., Nam E.S., Cho S.J., Rho Y.-S., Ahn H.Y. (2008). The Prevalence and Significance of Incidental Thyroid Nodules Identified on Computed Tomography. J. Comput. Assist. Tomogr..

[16] Shie P., Cardarelli R., Sprawls K., Fulda K.G., Taur A. (2009). Systematic review: Prevalence of malignant incidental thyroid nodules identified on fluorine-18 fluorodeoxyglucose positron emission tomography. Nucl. Med. Commun..

[17] Soelberg K.K., Bonnema S.J., Brix T.H., Hegedüs L. (2012). Risk of Malignancy in Thyroid Incidentalomas Detected by ^18^F-Fluorodeoxyglucose Positron Emission Tomography: A Systematic Review. Thyroid.

[18] Russ G., Bonnema S.J., Erdogan M.F., Durante C., Ngu R., Leenhardt L. (2017). European Thyroid Association Guidelines for Ultrasound Malignancy Risk Stratification of Thyroid Nodules in Adults: The EU-TIRADS. Eur. Thyroid. J..

[19] Haugen B.R., Alexander E.K., Bible K.C., Doherty G.M., Mandel S.J., Nikiforov Y.E., Pacini F., Randolph G.W., Sawka A.M., Schlumberger M. (2016). 2015 American Thyroid Association Management Guidelines for Adult Patients with Thyroid Nodules and Differentiated Thyroid Cancer: The American Thyroid Association Guidelines Task Force on Thyroid Nodules and Differentiated Thyroid Cancer. Thyroid.

[20] Shin J.H., Baek J.H., Chung J., Ha E.J., Kim J.-H., Lee Y.H., Lim H.K., Moon W.-J., Na D.G., Park J.S. (2016). Ultrasonography Diagnosis and Imaging-Based Management of Thyroid Nodules: Revised Korean Society of Thyroid Radiology Consensus Statement and Recommendations. Korean J. Radiol..

[21] Gharib H., Papini E., Garber J.R., Duick D.S., Harrell R.M., Hegedüs L., Paschke R., Valcavi R., Vitti P. (2016). American Association of Clinical Endocrinologists, American College of Endocrinology, and Associazione Medici Endocrinologi Medical Guidelines for Clinical Practice for the Diagnosis and Management of Thyroid Nodules—2016 Update Appendix. Endocr. Pract..

[22] Tessler F.N., Middleton W.D., Grant E.G., Hoang J.K., Berland L.L., Teefey S.A., Cronan J.J., Beland M.D., Desser T.S., Frates M.C. (2017). ACR Thyroid Imaging, Reporting and Data System (TI-RADS): White Paper of the ACR TI-RADS Committee. J. Am. Coll. Radiol..

[23] Zhou J., Yin L., Wei X., Zhang S., Song Y., Luo B., Li J., Qian L., Cui L., Chen W. (2020). 2020 Chinese guidelines for ultrasound malignancy risk stratification of thyroid nodules: The C-TIRADS. Endocrine.

[24] Hoang J.K., Asadollahi S., Durante C., Hegedüs L., Papini E., Tessler F.N. (2022). An International Survey on Utilization of Five Thyroid Nodule Risk Stratification Systems: A Needs Assessment with Future Implications. Thyroid.

[25] Durante C., Hegedüs L., Na D.G., Papini E., Sipos J.A., Baek J.H., Frasoldati A., Grani G., Grant E., Horvath E. (2023). International Expert Consensus on US Lexicon for Thyroid Nodules. Radiology.

[26] McHugh M.L. (2012). Interrater reliability: The kappa statistic. Biochem. Medica.

[27] Grani G., Lamartina L., Cantisani V., Maranghi M., Lucia P., Durante C. (2018). Interobserver agreement of various thyroid imaging reporting and data systems. Endocr. Connect..

[28] Persichetti A., Di Stasio E., Coccaro C., Graziano F.M., Bianchini A., Di Donna V., Corsello S.M., Valle D., Bizzarri G., Frasoldati A. (2020). Inter- and Intraobserver Agreement in the Assessment of Thyroid Nodule Ultrasound Features and Classification Systems: A Blinded Multicenter Study. Thyroid.

[29] Ha E.J., Chung S.R., Na D.G., Ahn H.S., Chung J., Lee J.Y., Park J.S., Yoo R.-E., Baek J.H., Baek S.M. (2021). 2021 Korean Thyroid Imaging Reporting and Data System and Imaging-Based Management of Thyroid Nodules: Korean Society of Thyroid Radiology Consensus Statement and Recommendations. Korean J. Radiol..

[30] Koh J., Kim S.-Y., Lee H.S., Kim E.-K., Kwak J.Y., Moon H.J., Yoon J.H. (2017). Diagnostic performances and interobserver agreement according to observer experience: A comparison study using three guidelines for management of thyroid nodules. Acta Radiol..

[31] Choi S.H., Kim E.-K., Kwak J.Y., Kim M.J., Son E.J. (2010). Interobserver and Intraobserver Variations in Ultrasound Assessment of Thyroid Nodules. Thyroid.

[32] Solymosi T., Hegedűs L., Bonnema S.J., Frasoldati A., Jambor L., Karanyi Z., Kovacs G.L., Papini E., Rucz K., Russ G. (2023). Considerable interobserver variation calls for unambiguous definitions of thyroid nodule ultrasound characteristics. Eur. Thyroid. J..

[33] Furuya-Kanamori L., Bell K.J., Clark J., Glasziou P., Doi S.A. (2016). Prevalence of Differentiated Thyroid Cancer in Autopsy Studies Over Six Decades: A Meta-Analysis. J. Clin. Oncol..

[34] Tappouni R.R., Itri J.N., McQueen T.S., Lalwani N., Ou J.J. (2019). ACR TI-RADS: Pitfalls, Solutions, and Future Directions. Radiographics.

[35] Jegerlehner S., Bulliard J.-L., Aujesky D., Rodondi N., Germann S., Konzelmann I., Chiolero A., NICER Working Group (2017). Overdiagnosis and overtreatment of thyroid cancer: A population-based temporal trend study. PLoS ONE.

[36] Wildman-Tobriner B., Ahmed S., Erkanli A., Mazurowski M.A., Hoang J.K. (2020). Using the American College of Radiology Thyroid Imaging Reporting and Data System at the Point of Care: Sonographer Performance and Interobserver Variability. Ultrasound Med. Biol..

[37] Kim S.H., Park C.S., Jung S.L., Kang B.J., Kim J.Y., Choi J.J., Kim Y.I., Oh J.K., Oh J.S., Kim H. (2010). Observer Variability and the Performance between Faculties and Residents: US Criteria for Benign and Malignant Thyroid Nodules. Korean J. Radiol..

[38] Beland M.D., Kwon L., Delellis R.A., Cronan J.J., Grant E.G. (2011). Nonshadowing Echogenic Foci in Thyroid Nodules. J. Ultrasound Med..

[39] Tahvildari A.M., Pan L., Kong C.S., Desser T. (2016). Sonographic-Pathologic Correlation for Punctate Echogenic Reflectors in Papillary Thyroid Carcinoma. J. Ultrasound Med..

[40] Piticchio T., Russ G., Radzina M., Frasca F., Durante C., Trimboli P. (2024). Head-to-head comparison of American, European, and Asian TIRADSs in thyroid nodule assessment: Systematic review and meta-analysis. Eur. Thyroid. J..

[41] Tong W.J., Wu S.H., Cheng M.Q., Huang H., Liang J.Y., Li C.Q., Guo H.L., He D.N., Liu Y.H., Xiao H. (2023). Integration of Artificial Intelligence Decision Aids to Reduce Workload and Enhance Efficiency in Thyroid Nodule Management. JAMA Netw. Open.

